# Amended engineered soil promotes coordinated soil-plant-microbiome responses during early-stage slope restoration

**DOI:** 10.3389/fbioe.2026.1918442

**Published:** 2026-09-03

**Authors:** Zhengfeng Chen, Yonghui Du, Hong Li, Xing Wen, Li Chen, Wenguo Wang, Peike Wu

**Affiliations:** 1 China Southwest Geotechnical Investigation and Design Institute Co. Ltd., Chengdu, China; 2 College of Ecology and Environment, Chengdu University of Technology, Chengdu, China; 3 Key Laboratory of Development and Application of Rural Renewable Energy, Biogas Institute of Ministry of Agriculture and Rural Affairs, Chengdu, China

**Keywords:** amended engineered soil, slope ecological restoration, soil-plant-microbiome interactions, substrate reconstruction, *Lolium perenne*

## Abstract

Amended Engineered Soil (AES), a functional substrate incorporating biomass-derived straw fiber, has been applied for the ecological restoration of degraded slopes. However, how AES affects the coordinated responses of substrate structure, root development, and rhizosphere microbiome assembly during early-stage restoration remains unclear. In this study, AES and Conventional Engineered Soil (CES) were compared during a 90-day slope restoration experiment using *Lolium perenne* as the target species. Compared with CES, AES substantially improved substrate moisture and nutrient conditions, with water content increasing from approximately 10.4%–16.6% and available phosphorus increasing more than fivefold. AES also promoted the formation of large water-stable aggregates, indicating improved structural stability of the reconstructed substrate. These changes were accompanied by marked improvements in vegetation establishment, including a 18.4% increase in plant height, more than 40% increase in dry biomass, and a pronounced increase in root length. Root tensile strength was also enhanced, suggesting greater belowground reinforcement. AES significantly increased bacterial richness and Shannon diversity, whereas the fungal response was mainly reflected in increased richness rather than diversity. Correlation analysis further showed close associations among substrate nutrient conditions, plant growth, root reinforcement, and microbial diversity. These findings suggest that AES promotes early-stage slope restoration through the coordinated improvement of substrate quality, vegetation establishment, root development, and rhizosphere microbial communities, while providing a sustainable pathway for biomass resource utilization.

## 1 Introduction

Slope ecosystems associated with transportation, hydraulic, and mining engineering are frequently subjected to large-scale excavation and backfilling activities, which severely disturb native soil-vegetation systems (Cao et al., 2023; Fang et al., 2024; Liu et al., 2025). Such disturbances commonly induce substrate compaction, structural instability, nutrient depletion, and the loss of belowground microbial habitats, thereby weakening erosion resistance and constraining natural vegetation recovery (Wen et al., 2021; Duan et al., 2026). Under these conditions, the failure to rapidly reconstruct substrate functions often results in persistent ecological instability and low restoration efficiency. Consequently, restoring both physical substrate quality and biological establishment has become a major challenge in slope ecological engineering.

Engineered soil replacement is widely adopted to improve substrate conditions during slope restoration (Porter et al., 2026). In practical applications, restoration substrates are generally categorized as Conventional Engineered Soil (CES) and Amended Engineered Soil (AES) (Moreno-Mateos et al., 2012). Compared with CES, AES is typically reconstructed through optimization of particle composition, pore architecture, nutrient availability, and the incorporation of organic or functional additives to create a more favorable environment for vegetation establishment (Porter et al., 2026). Rather than functioning solely as a plant growth medium, AES may also act as an engineered ecological substrate capable of regulating root development, microbial colonization, and rhizosphere habitat formation during ecosystem recovery (Lehmann et al., 2020; Romero-Estonllo et al., 2023).

Previous studies on engineered soils or organic amendments have mainly focused on soil fertility improvement, substrate stabilization, or vegetation establishment alone (Ayangbenro et al., 2022; Li et al., 2024). However, slope restoration is not only a process of improving soil properties or increasing vegetation cover, but also involves coordinated responses among substrate structure, root development, and belowground microbial communities. Substrate porosity, aggregate stability, and nutrient availability can influence root penetration and microbial habitat formation, while root growth and microbial activity may further contribute to substrate stabilization and nutrient turnover. Therefore, an integrated evaluation of substrate structure, plant performance, root traits, and microbial responses is needed to better understand the ecological effects of engineered substrates during early-stage slope restoration.

Physical substrate conditions play a particularly important role during early-stage slope restoration because they directly influence root penetration, water retention, oxygen diffusion, nutrient transport, and microbial habitat heterogeneity. Improvements in pore continuity and aggregate stability may facilitate root establishment and microbial assembly, whereas plant-derived carbon inputs and microbial activities subsequently contribute to aggregate formation and nutrient cycling (Van Der Heijden et al., 2008; Fierer, 2017). Therefore, slope restoration should be regarded as a coupled ecological assembly process involving simultaneous reconstruction of substrate structure, vegetation establishment, and microbiome development, rather than the independent improvement of soil or plant components alone.

In this study, CES and AES were compared under field slope conditions using *L. perenne* as the target restoration species. By integrating substrate physicochemical properties, aggregate stability, plant growth, root reinforcement traits, and microbial community characteristics, this study aimed to clarify how AES promotes coordinated soil–plant–microbiome responses during early-stage slope restoration.

## 2 Materials and methods

### 2.1 Study area

The field experiment was conducted on a highway rock slope in Guiyang City, Guizhou Province, China (106°27′–107°13′ E, 26°11′–26°55′ N), at an average elevation of 1071 m. The region is characterized by a subtropical humid monsoon climate with a mean annual temperature of 14.5 °C and annual precipitation of approximately 1500 mm. The experimental slope had an inclination of approximately 25°, and the parent material consisted primarily of limestone-derived weathered soil, representing typical degraded slope conditions in the region.

### 2.2 Experimental design

#### 2.2.1 Soil preparation

A single-factor randomized block design was employed to evaluate the effects of engineered substrate reconstruction on slope ecological restoration. Two engineered soil treatments were established in the field experiment: Conventional Engineered Soil (CES) and Amended Engineered Soil (AES). Both treatments were prepared using the same batch of local surface soil collected from the 0–20 cm layer of the study area to ensure a consistent geological background. Before substrate preparation, the collected soil was air-dried, homogenized, and manually cleaned to remove visible plant residues, roots, gravel, and stones larger than 5 mm.

For CES, the pretreated local soil was directly used as the conventional engineered substrate without additional functional amendments. For AES, the same pretreated local soil was used as the base material (20 kg m^-2^) and reconstructed by incorporating functional additives functional additives included corn straw-derived fiber (450 g m^-2^), soil stabilizer (100 g/·m^-2^), water-retaining agent (200 g/·m^-2^), and compound fertilizers (N + P2O3+K2O≥40%(20-12-8)). These materials were thoroughly mixed with the pretreated local soil before backfilling. Corn straw-derived fiber was used as an organic structural amendment to improve substrate structure and facilitate aggregate formation, while also enabling the reuse of agricultural residue reuse.

#### 2.2.2 Plot establishment and vegetation cultivation

A total of 20 experimental plots (4 m × 4 m) were established on the slope surface. Concrete barriers (30 cm in height, with 15 cm embedded into the rock surface) were constructed around each plot to prevent substrate loss and cross-contamination among treatments. Engineered substrates were backfilled to a uniform thickness of 15 cm, and substrate bulk density was controlled within 1.3–1.4 g cm^-3^ through standardized compaction procedures. *L. perenne* was selected as the target restoration species, given its rapid establishment, fibrous root system, and common use in slope revegetation and erosion control, and sown at a density of 15 g m^-2^. The 90-day period was selected as an early-stage restoration window to evaluate the initial effects of substrate reconstruction on vegetation establishment, root development, and microbial community responses. During this period, all plots were maintained under identical management conditions, and manual weeding was conducted when necessary.

### 2.3 Sample collection and analysis

#### 2.3.1 Soil sample collection and analysis

Soil samples were collected 90 days after sowing using an S-shaped sampling strategy. Five sampling points were established within each plot, and soil from the 0–15 cm layer was collected and composited into a single representative sample. Samples were transported to the laboratory under cooled conditions. After removing visible roots and gravel, one portion of each sample was air-dried for physicochemical analysis, while another portion was stored at −80 °C for microbial community analysis. Bulk density and total porosity were measured using the core sampling method. Water-stable macroaggregates (>0.25 mm) were quantified by wet sieving, and water retention capacity was evaluated gravimetrically. Soil organic matter was analyzed using potassium dichromate oxidation with external heating. Alkaline hydrolyzable nitrogen was determined using the alkaline diffusion method, available phosphorus was measured by the molybdenum-antimony colorimetric method, and available potassium was quantified using flame photometry.

#### 2.3.2 Plant sample collection and determination

Twenty *L. perenne* individuals were randomly sampled from each plot as subsamples for plant trait measurements. The mean value of the 20 subsampled plants within each plot was calculated and used as one plot-level replicate for statistical analysis. Plant height was measured using a steel ruler with 0.1 cm precision. Fresh biomass was determined immediately after removing surface moisture, while dry biomass was obtained after oven drying at 80 °C to constant weight. Root-to-shoot ratio was subsequently calculated. Root systems were carefully excavated and rinsed with deionized water prior to root length measurement. Root tensile strength was determined for roots with diameters ranging from 1.0 to 1.5 mm using a WDW-5E universal testing machine. Tensile strength was calculated according to De Baets et al., 2008 as follows:

$$\text{Tensile strength }\left(\text{MPa}\right)=\text{maximum tensile force }\left(N\right)/\text{cross}-\text{sectional area }\left({\text{mm}}^{2}\right)$$

#### 2.3.3 Microbial community analysis

Total soil microbial DNA was extracted using the CTAB method. The V4-V5 region of the bacterial 16S rRNA gene and the fungal ITS1 region were amplified by PCR. High-throughput sequencing was performed on an Illumina MiSeq platform. Sequence processing and microbial community analyses were conducted using QIIME2. Alpha diversity was evaluated using the Shannon and Chao1 indices, while community composition was analyzed at both phylum and genus levels.

### 2.4 Statistical analysis

All data are presented as mean ± standard deviation (SD). Statistical analyses were conducted using SPSS 26.0 software. Differences between treatments were evaluated by one-way analysis of variance (ANOVA), followed by the least significant difference (LSD) test at P < 0.05. Figures were generated using Origin 2021, and preliminary data processing was performed in Microsoft Excel 2019.

## 3 Results

### 3.1 AES reconstructs substrate physical structure and nutrient conditions

**FIGURE 1:**
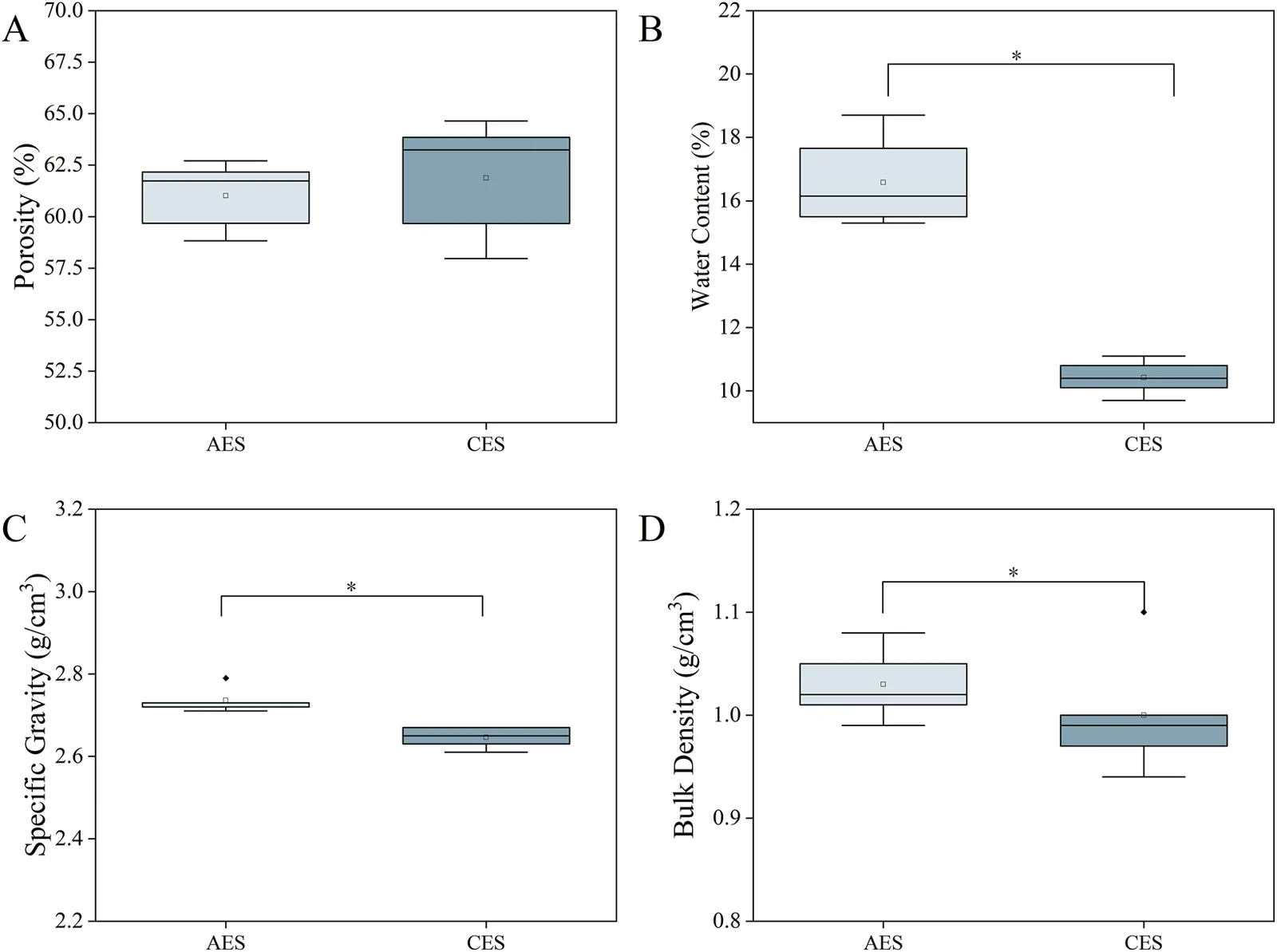
Physicochemical properties of Amended Engineered Soil (AES), and Conventional Engineered Soil (CES): **(A)** porosity; **(B)** water content; **(C)** specific gravity; and **(D)** bulk density. * indicates significant differences at *P* < 0.05.

**FIGURE 2:**
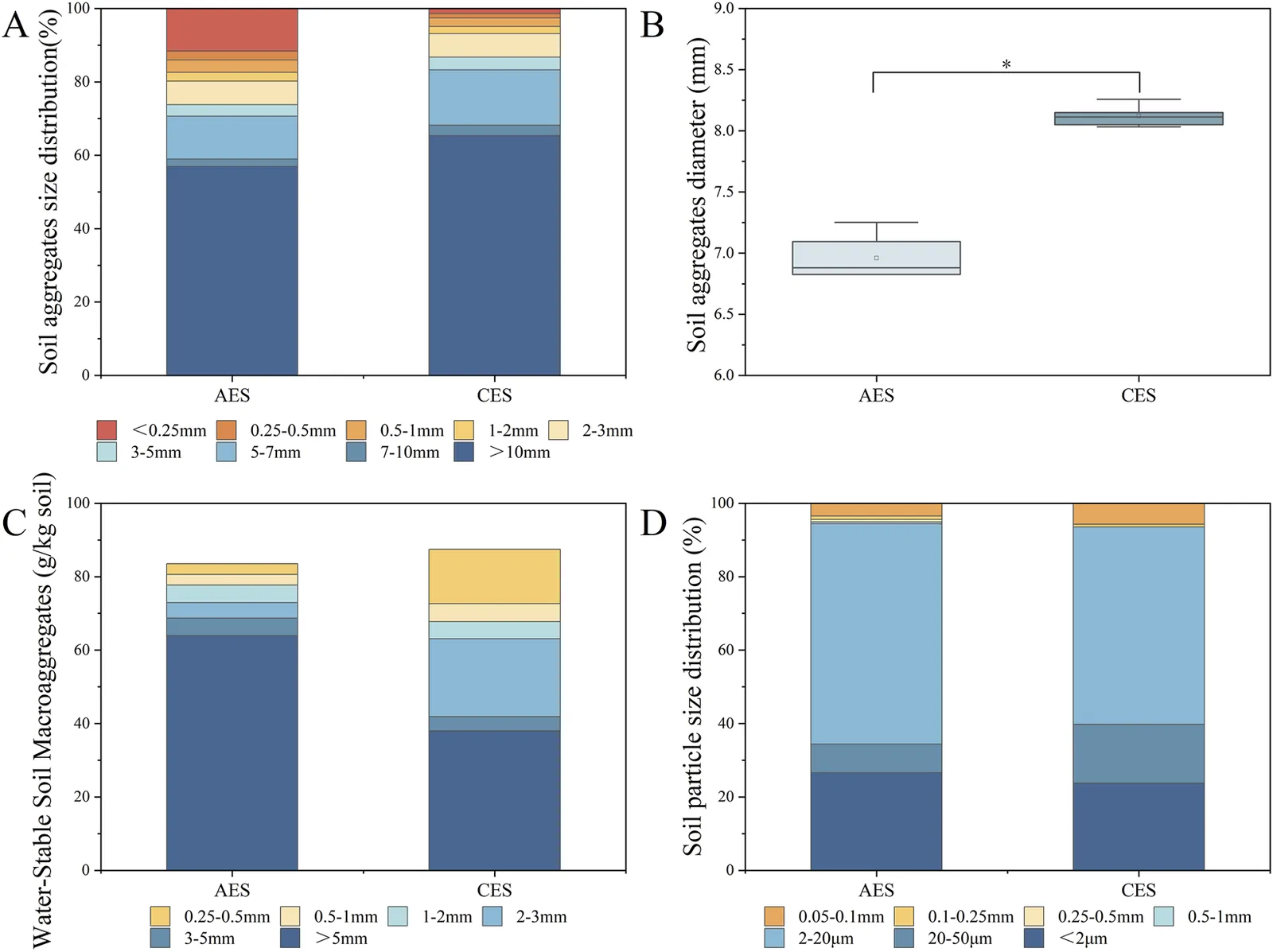
Aggregate and particle size characteristics of Amended Engineered Soil (AES) and Conventional Engineered Soil (CES): **(A)** aggregate size distribution; **(B)** aggregate diameter; **(C)** water-stable macroaggregate content; and **(D)** particle size distribution. * indicates significant differences at *P* < 0.05.

Significant differences in substrate physicochemical properties were observed between AES and CES after 90 days of restoration (*P* < 0.05; Figures 1, 2). Compared with CES, AES showed a slightly lower porosity, whereas its water content increased markedly from approximately 10.4%–16.6%. Particle density and bulk density were also slightly higher in AES. These results suggest that AES substantially improved substrate moisture status, although this improvement was not accompanied by increased total porosity or reduced bulk density.

Aggregate-size composition also differed between the two engineered substrates. Large aggregates constituted the dominant fraction in both treatments, but CES exhibited a greater mean aggregate diameter than AES. In contrast, AES contained a higher proportion of fine aggregates and a greater amount of water-stable aggregates larger than 5 mm, which increased from approximately 38–64 g kg^-1^ soil. These changes indicate that AES redistributed aggregate-size fractions and enhanced the stability of the large-aggregate fraction rather than uniformly increasing aggregate size. Particle-size distributions were broadly similar between treatments and were dominated by particles within the 2–20 μm range.

AES also markedly altered substrate nutrient conditions (Table 1). Organic matter content increased from 2.57 ± 0.91 g kg^-1^ in CES to 21.26 ± 1.49 g kg^-1^ in AES, while available phosphorus increased from 9.55 ± 0.38 to 52.80 ± 5.39 mg kg^-1^. Total phosphorus and available potassium were also significantly higher in AES, whereas total nitrogen was slightly lower than that in CES. Overall, AES primarily improved substrate moisture, organic matter accumulation, phosphorus availability, and large water-stable aggregate stability, thereby creating a more favorable substrate environment for early vegetation establishment.

**TABLE 1:** Physical and chemical properties of soil.

Parameter	Amended engineered soil	Conventional engineered soil
Organic matter/(g·kg^-1^)	21.26 ± 1.49a	2.57 ± 0.91b
TN/(g·kg^-1^)	0.47 ± 0.05b	0.63 ± 0.13a
TP/(g·kg^-1^)	4.65 ± 0.25a	2.69 ± 0.29b
Available phosphorus/(mg·kg^-1^)	52.80 ± 5.39a	9.55 ± 0.38b
Available potassium/(mg·kg^-1^)	5.83 ± 0.61a	4.29 ± 0.51b

Different lowercase letters within the same row indicate significant differences between treatments at *P* < 0.05 according to the LSD, test.

### 3.2 AES promotes vegetation establishment and root reinforcement

**FIGURE 3:**
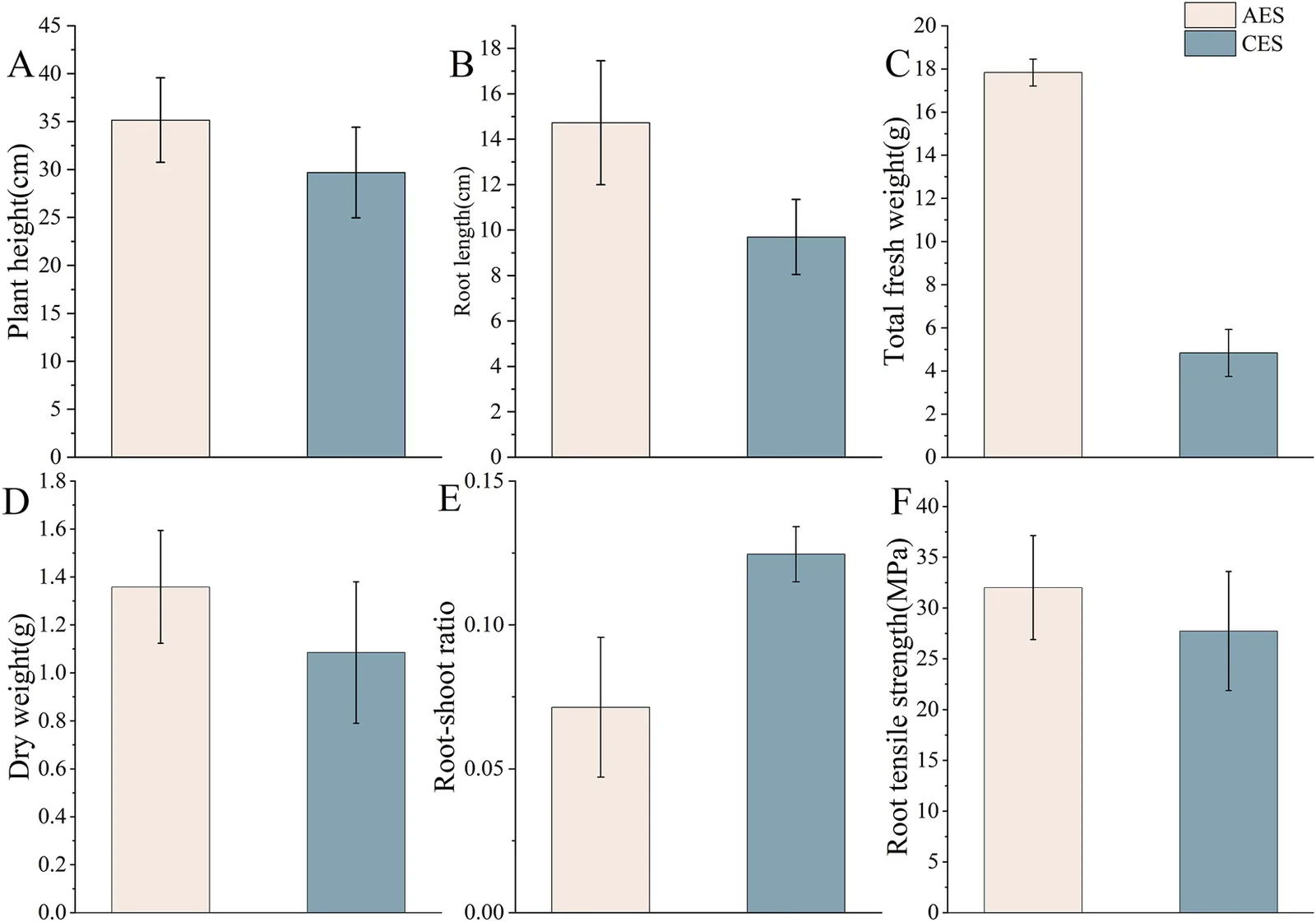
**(A)** Plant height; **(B)** Root length; **(C)** Total fresh weight; **(D)** Dry weight; **(E)** Root-shoot ratio; **(F)** Root tensile strength. AES, Amended engineered soil; CES, Conventional engineered soil.

Substantial differences in plant growth and root development were observed between treatments after 90 days of cultivation (Figure 3). *L. perenne* grown in AES exhibited significantly greater aboveground and belowground development than plants grown in CES. Plant height increased from 29.7 ± 4.7 cm in CES to 35.15 ± 4.4 cm in AES, accompanied by pronounced increases in fresh and dry biomass. Fresh biomass under AES reached 17.8 ± 0.6 g, more than double that measured in CES (4.8 ± 1.0 g), while dry biomass increased from 1.0 ± 0.2 g in CES to 1.4 ± 0.2 g in AES. The root-to-shoot ratio was lower in AES than in CES, indicating that the increase in aboveground biomass was proportionally greater than the increase in belowground biomass. Root traits exhibited similar trends. Root length in AES reached 14.7 ± 2.7 cm, whereas roots in CES averaged only 9.7 ± 1.7 cm. Root tensile strength was also higher in AES (32.0 ± 5.1 MPa) than in CES (27.7 ± 5.8 MPa), suggesting improved mechanical reinforcement capacity within the restored substrate.

### 3.3 AES alters microbial diversity and community composition

**FIGURE 4:**
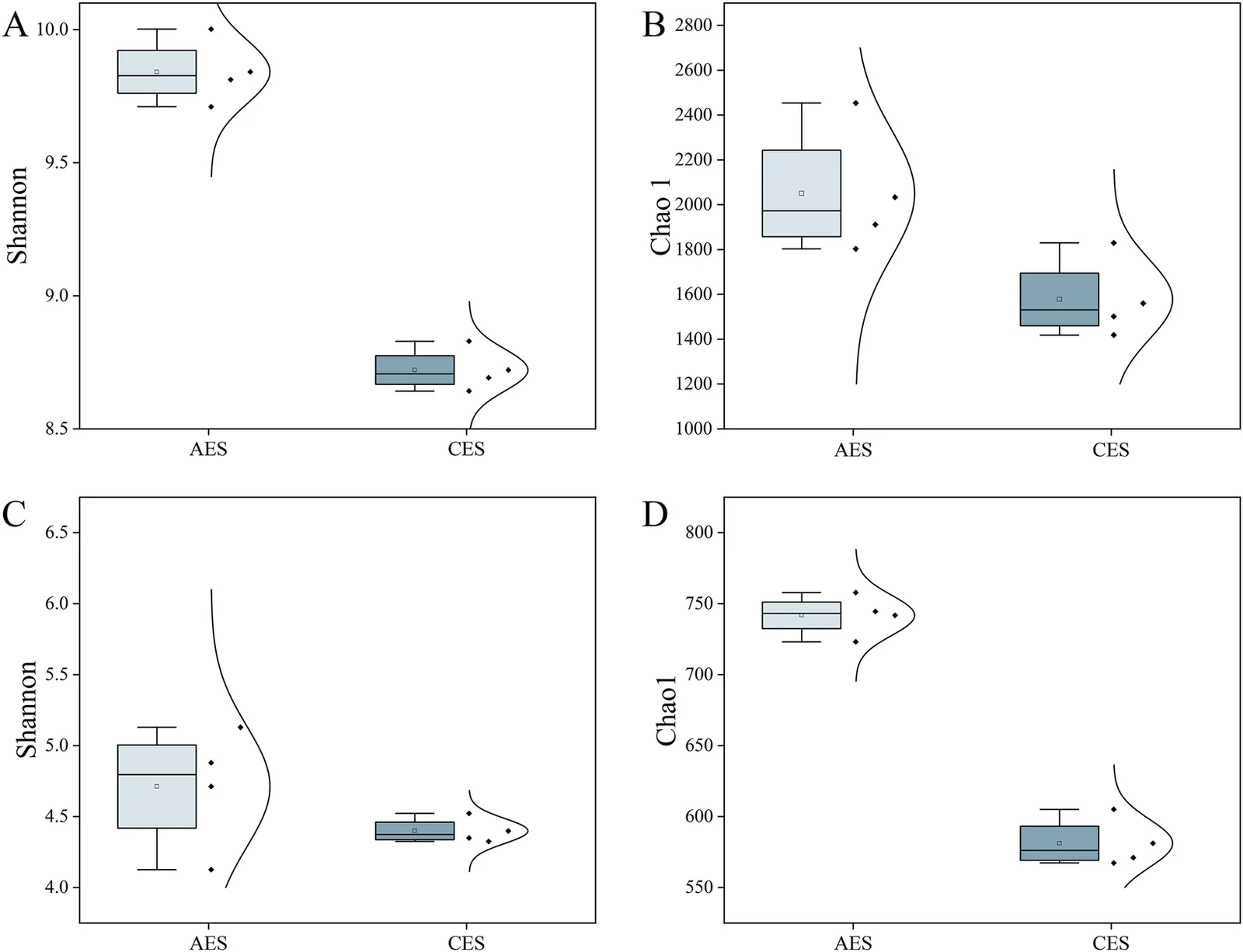
Alpha Diversity of Bacterial (**(A)** Shannon Index; **(B)** Chao1 Index) and Fungal (**(C)** Shannon Index; **(D)** Chao1 Index) Communities inAmended Engineered Soil and Conventional Engineered Soil.

Alpha-diversity patterns differed between bacterial and fungal communities (Figure 4). Bacterial richness, represented by the Chao1 index, was higher in AES (2050.35 ± 284.81) than in CES (1577.38 ± 177.87). Similarly, the bacterial Shannon index increased from 8.72 ± 0.08 in CES to 9.84 ± 0.12 in AES. Fungal Chao1 richness was also higher in AES (741.74 ± 14.27) than in CES (581.13 ± 16.99), whereas fungal Shannon diversity did not differ significantly between AES (4.71 ± 0.43) and CES (4.40 ± 0.09). PCoA analysis further showed clear separation of bacterial and fungal communities between AES and CES treatments (Supplementary Figure S2), indicating differences in microbial community composition after substrate amendment.

**FIGURE 5:**
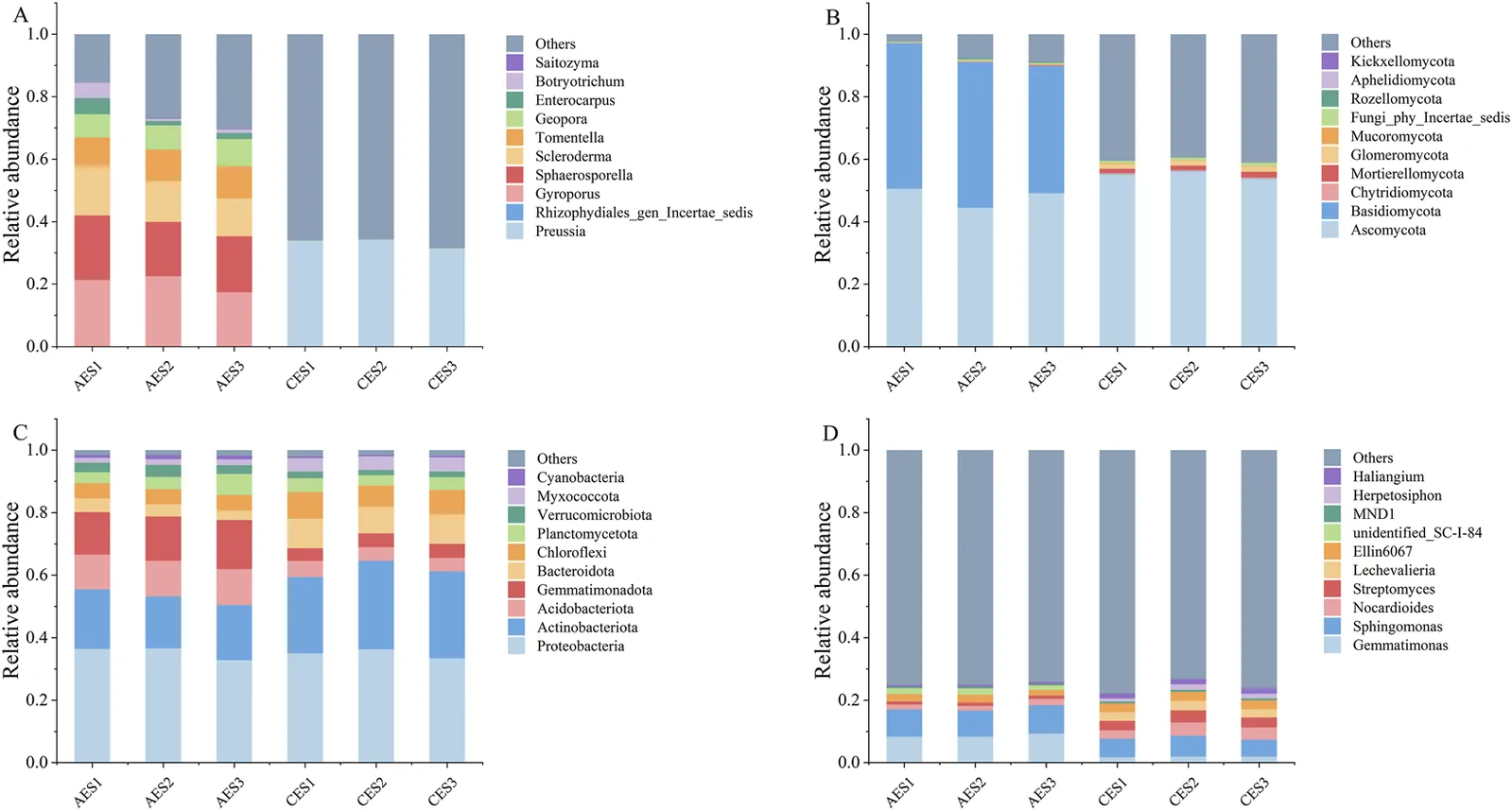
Relative Abundance of Fungal (**(A)** phylum Level; **(B)** genus Level) and Bacterial (**(C)** phylum Level; **(D)** genus Level) Communities in Amended Engineered Soil and Conventional Engineered Soil.

**FIGURE 6:**
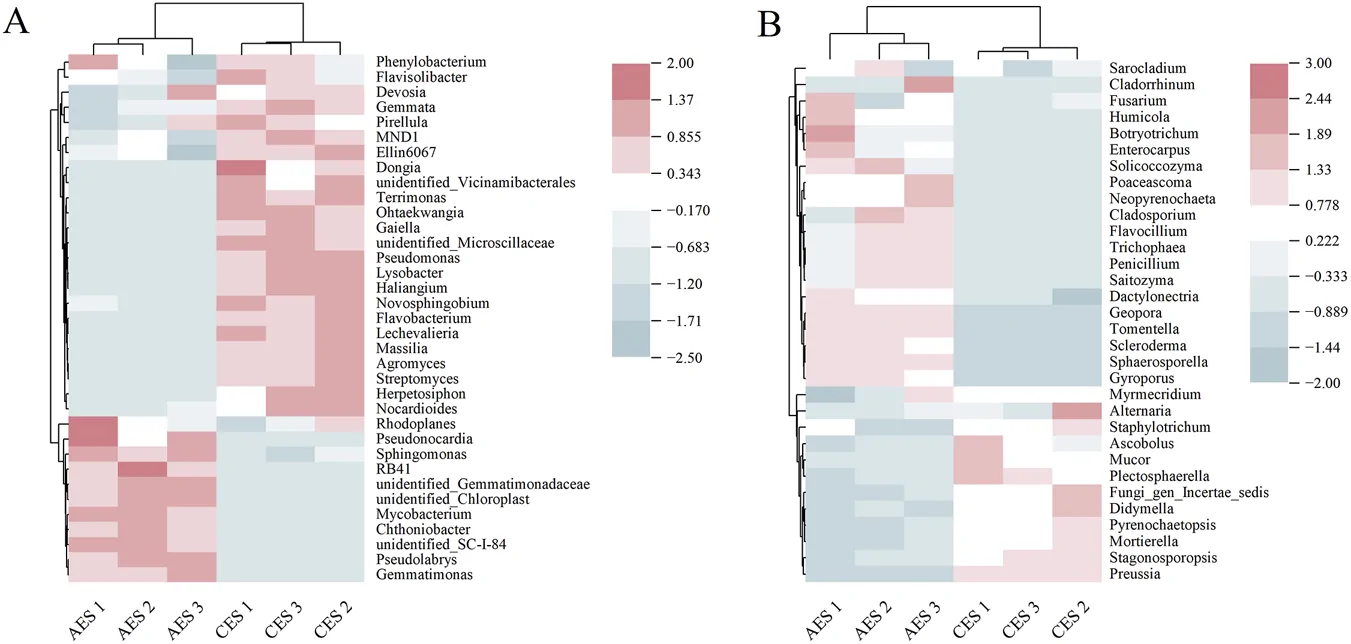
Heatmap of bacterial **(A)** and fungal **(B)** genus-level communities in Amended engineered soil and conventional engineered soil.

Marked differences in microbial community composition were observed between AES and CES treatments (Figure 5). At the phylum level, bacterial communities showed distinct distribution patterns between treatments, with AES exhibiting relatively higher proportions of Actinobacteriota, whereas CES showed greater proportions of Proteobacteria. For fungal communities, AES displayed relatively higher abundances of Basidiomycota, while Ascomycota represented a dominant fungal phylum in both treatments with different relative contributions.At the genus level, several dominant bacterial and fungal taxa exhibited different distribution patterns between AES and CES. In bacterial communities, *Streptomyces*, Sphingomonas, and Gemmatimonas showed higher relative abundances under AES, whereas Nocardioides showed relatively higher abundance under CES. For fungal communities, genera including Preussia, Gyroporus, and Sphaerosporella showed different relative abundances between treatments. Heatmap analysis further revealed distinct clustering patterns of dominant bacterial and fungal genera between AES and CES, indicating differences in microbial community composition following substrate amendment (Figure 6). Overall, AES altered the taxonomic composition and distribution patterns of dominant bacterial and fungal communities during early-stage slope restoration.

**FIGURE 7:**
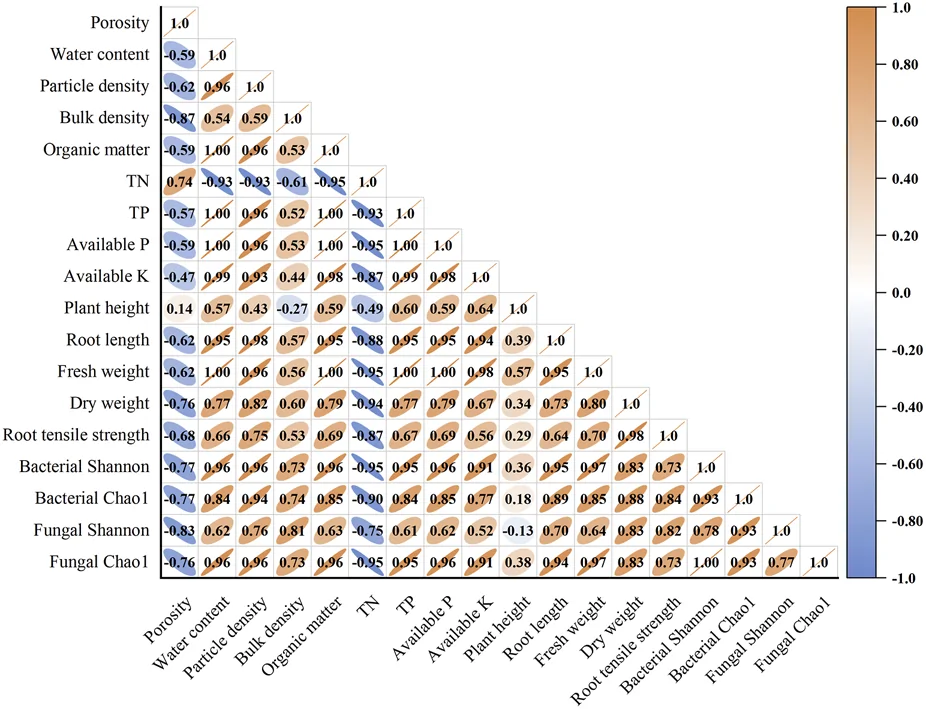
Correlation heatmap of key indicators in the soil-plant-microbiome system.

### 3.4 Coordinated responses among soil, plant, and microbial variables

Correlation analysis showed that organic matter, total phosphorus, available phosphorus, and available potassium were positively associated with several plant growth and microbial diversity indicators (Figure 7). Organic matter and available phosphorus were significantly correlated with plant height, root length, dry biomass, bacterial Shannon diversity, and fungal Chao1 richness (P < 0.05). In contrast, porosity showed no significant relationships with the principal plant or microbial indicators. Bulk density was not significantly associated with most plant traits but was positively correlated with bacterial Shannon diversity and fungal Chao1 richness.

## 4 Discussion

### 4.1 Reconstruction of physical substrate niches during early-stage slope restoration

The improved restoration performance under AES was associated with changes in substrate moisture status, aggregate composition, and nutrient availability rather than a simple reduction in substrate density. Compared with CES, AES maintained substantially higher water content and contained a greater amount of water-stable aggregates larger than 5 mm. However, AES exhibited slightly lower porosity and higher bulk density, indicating that the amendments did not produce a uniformly looser substrate structure. Instead, the incorporated straw fiber, water-retaining agent, and organic amendments may have enhanced water adsorption and promoted the formation or preservation of stable large aggregates (Zhang et al., 2014; Zhang et al., 2023; Redmile-Gordon et al., 2020).

Water availability is particularly important during early-stage slope restoration because shallow reconstructed substrates are susceptible to rapid drying and strong fluctuations in moisture conditions. The higher water content under AES may therefore have prolonged water availability for root growth and microbial activity. Meanwhile, the increase in large water-stable aggregates suggests improved resistance of the substrate to wetting-induced disintegration and particle detachment. The substantial enrichment of organic matter may have further contributed to aggregate stabilization by providing binding materials and carbon substrates for microbial activity (Li et al., 2021). Thus, the principal physical effect of AES was better interpreted as enhanced moisture maintenance and redistribution of aggregate fractions rather than generalized substrate loosening.

### 4.2 Root-mediated reinforcement and vegetation establishment under AES

The enhanced plant performance under AES was more likely associated with improved moisture availability, nutrient supply, and aggregate stability than with reduced substrate density. Higher water content may have alleviated short-term water limitation, while the pronounced increases in organic matter and available phosphorus provided more favorable conditions for biomass accumulation and root extension. The greater abundance of large water-stable aggregates may also have created more persistent structural units for root anchorage (Gyssels et al., 2005; Loades et al., 2010). These combined changes likely supported both aboveground growth and the development of a more extensive root reinforcement network.

The improved moisture and nutrient conditions under AES likely supported sustained root elongation and belowground biomass allocation facilitated root extension into deeper substrate layers. At the same time, greater water retention and nutrient availability may have supported sustained biomass accumulation and belowground carbon allocation (Lynch, 2011; Freschet et al., 2021). Beyond their structural role, developing root systems may also contribute to ecological stabilization through rhizosphere-mediated processes. Root exudates and litter inputs provide carbon substrates for microbial metabolism, while root penetration physically modifies substrate structure and promotes aggregate formation (Kuzyakov and Razavi, 2019; Lehmann et al., 2020). Consequently, root development under AES likely contributed simultaneously to substrate stabilization, rhizosphere activation, and progressive ecosystem recovery.

### 4.3 Microbial responses to substrate reconstruction and rhizosphere development

Microbial communities showed distinct responses to substrate reconstruction during early-stage restoration. Compared with CES, AES significantly increased bacterial Chao1 and Shannon indices, indicating simultaneous improvements in bacterial richness and overall diversity. This response was likely related to the higher water content, organic matter, and available phosphorus under AES, which provided greater resource availability and more favorable microsites for bacterial colonization. Soil bacterial communities are highly sensitive to changes in moisture, nutrient supply, and habitat heterogeneity, particularly in recently reconstructed substrates where environmental constraints strongly regulate community assembly (Fierer, 2017; Delgado-Baquerizo et al., 2020).

Fungal communities exhibited a different response pattern. AES significantly increased fungal Chao1 richness, whereas the increase in fungal Shannon diversity was not significant. This divergence suggests that AES facilitated the recruitment or establishment of additional fungal taxa but did not substantially improve community evenness within the 90-day restoration period. Compared with bacteria, fungal community development may depend more strongly on the gradual establishment of stable rhizosphere niches, continuous root-derived carbon inputs, and the accumulation of plant residues. Consequently, fungal richness may respond relatively rapidly to improved substrate conditions, whereas the recovery of community structure and evenness may require a longer period.

PCoA also showed clear separation of bacterial and fungal communities between AES and CES, indicating that substrate amendment was associated with substantial shifts in microbial community composition. These shifts were likely driven by the combined effects of altered moisture conditions, nutrient availability, organic inputs, and root development, all of which can influence microbial habitat selection and community assembly (Jiao et al., 2018; Trivedi et al., 2020). Overall, AES promoted bacterial diversity and fungal richness, but bacterial and fungal communities followed different recovery trajectories during early-stage slope restoration.

### 4.4 Coordinated soil-plant-microbiome interactions during ecosystem recovery

The correlation pattern suggests that the restoration effect of AES was driven mainly by improved resource availability rather than by changes in bulk physical structure. Organic matter and available phosphorus were closely associated with plant growth and microbial diversity, indicating that the substantial enrichment of carbon and phosphorus created more favorable conditions for root development and rhizosphere colonization. Organic matter can improve nutrient retention and provide carbon substrates for microbial activity, while phosphorus availability supports root elongation and biomass formation (Lynch, 2011; Lehmann et al., 2020). Plant development and rhizosphere microbial communities are also closely linked because root-derived carbon inputs support microbial colonization, while microbial processes contribute to nutrient turnover and plant establishment(Van Der Heijden et al., 2008; Trivedi et al., 2020).In contrast, porosity showed no clear relationship with the main biological indicators, while bulk density was not consistently associated with plant performance. This explains why AES still supported better vegetation and microbial development despite its slightly lower porosity and higher bulk density. The result suggests that moisture maintenance, nutrient availability, and aggregate stability were more important than generalized substrate loosening during the early restoration stage.

Root tensile strength was also positively related to several plant and microbial indicators, suggesting that root reinforcement developed together with biomass accumulation and rhizosphere establishment. Greater root development can strengthen particle binding and increase rhizosphere carbon inputs, thereby linking mechanical stabilization with biological recovery(Gyssels et al., 2005; Kuzyakov and Razavi, 2019). Overall, these relationships indicate that the early restoration advantage of AES arose from the combined effects of resource enrichment, moisture maintenance, and root development.

## 5 Conclusion

This study demonstrated that Amended Engineered Soil (AES) effectively promoted early-stage ecological restoration of degraded slopes by improving substrate quality and supporting the coordinated development of vegetation and microbial communities. Compared with CES, AES markedly increased substrate water content, organic matter, phosphorus availability, and the abundance of large water-stable aggregates, indicating that its main effect was to enhance moisture conservation, nutrient supply, and aggregate stability rather than simply loosening the substrate.These improvements were accompanied by greater plant height, biomass accumulation, root elongation, and root tensile strength, suggesting that AES strengthened both vegetation establishment and root-mediated slope reinforcement. AES also increased bacterial richness and diversity and promoted fungal richness, although the recovery of fungal community diversity appeared to be slower during the 90-day restoration period. The correlations among substrate properties, plant traits, and microbial indicators further reflected synchronous responses of the soil–plant–microbiome system to substrate reconstruction.Overall, AES created a more favorable ecological substrate for early slope restoration by integrating improved moisture and nutrient conditions, enhanced structural stability, root development, and microbial community recovery.

## Data Availability

The data presented in the study are deposited in the NCBI Sequence Read Archive (SRA) repository, accession number PRJNA1518407. The mandatory sequencing data have been deposited to NCBI SRA under BioProject ID PRJNA1518407. We confirm the completion of data deposition, and the data will be publicly released when the article is published.
