# Supplementary material for: Amended engineered soil promotes coordinated soil-plant-microbiome responses during early-stage slope restoration
Source: Front Bioeng Biotechnol. 2026 Sep 3;14:1918442. doi: 10.3389/fbioe.2026.1918442 (PMC13582654; doi:10.3389/fbioe.2026.1918442)
Supplement: Supplementary file 1 [file Supplementaryfile1.docx]

Supplementary Fig. S1. Schematic diagram of the experimental plot layout on the slope surface.

Supplementary Fig. S2. Principal Coordinate Analysis (PCoA) of Bacterial (A) and Fungal (B) Community Structure in Amended Engineered Soil, and Conventional Engineered Soil.
